# Supplementary material for: Patient, hospital and country-level risk factors of all-cause mortality among patients with chronic heart failure: Prospective international cohort study
Source: PLoS One. 2021 May 10;16(5):e0250931. doi: 10.1371/journal.pone.0250931 (PMC8109791; doi:10.1371/journal.pone.0250931)
Supplement: S1 Appendix — (DOCX) [file pone.0250931.s001.docx]

**Supporting information for “Patient, hospital and country-level risk factors of all-cause mortality among patients with chronic heart failure: Prospective international cohort study”**

**S1 Table.** Sample selection process by country.

|  | **1) Baseline Patients** | **2) Patients whose country is included in ATLAS** | **3) Patients whose country is included in ATLAS & admission hospital tracked** | **4) Patients with info on admission hospital & country included in ATLAS & available at follow up** | **5) Patients with info on admission hospital & country included in ATLAS & available at follow up & more than 30 obs per country** |
| --- | --- | --- | --- | --- | --- |
| Total | 37 countries, 14742 patients | 30 countries, 13,656 patients | 27 countries, 11,347patients | 27 countries, 9320patients | 22 countries, 9,277patients |
| Argentina | 410 |  |  |  |  |
| Austria | 216 | 216 | 216 | 171 | 171 |
| Belarus | 61 | 61 | 61 | 59 | 59 |
| Bosnia & Herzegovina | 51 | 51 | 51 | 39 | 39 |
| Bulgaria | 92 | 92 | 90 | 63 | 63 |
| Croatia | 13 | 13 | 13 | 3 |  |
| Cyprus | 141 | 141 | 141 | 66 | 66 |
| Czech Republic | 574 | 574 | 495 | 454 | 454 |
| Denmark | 295 | 295 | 90 | 76 | 76 |
| Egypt | 689 | 689 | 689 | 537 | 537 |
| Estonia | 81 | 81 | 68 | 68 | 68 |
| Finland | 10 | 10 |  |  |  |
| France | 601 | 601 | 492 | 441 | 441 |
| Georgia | 31 | 31 | 28 | 6 |  |
| Greece | 202 | 202 | 201 | 156 | 156 |
| Hungary | 620 | 620 | 238 | 212 | 212 |
| Israel | 499 | 499 | 499 | 485 | 485 |
| Italy | 1,967 | 1,967 | 1,495 | 1,321 | 1,321 |
| Kosovo | 93 |  |  |  |  |
| Kyrgyzstan | 176 |  |  |  |  |
| Latvia | 39 | 39 |  |  |  |
| Lithuania | 393 | 393 | 393 | 334 | 334 |
| Macedonia | 56 |  |  |  |  |
| Malta | 89 |  |  |  |  |
| Moldova | 19 | 19 | 19 | 13 |  |
| Poland | 433 | 433 | 324 | 294 | 294 |
| Portugal | 966 | 966 | 780 | 622 | 622 |
| Romania | 162 | 162 | 2 |  |  |
| Saudi Arabia | 100 |  |  |  |  |
| Serbia | 107 | 107 | 107 | 69 | 69 |
| Slovakia | 271 | 271 | 271 | 234 | 234 |
| Slovenia | 371 | 371 | 108 | 93 | 93 |
| Spain | 4,290 | 4,290 | 4,290 | 3,360 | 3,360 |
| Sweden | 276 | 276 |  |  |  |
| Switzerland | 44 | 44 | 44 | 21 |  |
| Turkey | 142 | 142 | 142 | 123 | 123 |
| Uruguay | 162 |  |  |  |  |

**S2 Table.** Baseline characteristics of the study population, hospitals and countries. Values are number (%) of total sample (N=14,742) unless stated otherwise.

| **Variable** | **Observed** | **Missing values** |
| --- | --- | --- |
| Female sex | 4285 (28.9) | 82 (0.6) |
| Age in years, mean (standard deviation) | 65.4 (13.1) | 189 (1.3) |
| BMI, mean (standard deviation) | 28.1 (5.1) | 502 (3.4) |
| BMI <20 | 104 (0.7) |  |
| BMI 20-24.9 | 3905 (26.4) |  |
| BMI 25-29.9 | 5839 (39.4) | 502 (3.4) |
| BMI 30-34.9 | 3158 (21.3) |  |
| BMI>35 | 1311 (8.9) |  |
| Systolic pressure, mean (standard deviation) | 124.2 (21) | 130 (0.9) |
| Ischemic Aetiology | 6387 (43.1) | 143 (1) |
| AF history | 5494 (37.1) | 91 (0.6) |
| Diabetes history | 4655 (31.4) | 84 (0.6) |
| PAD | 1698 (11.5) | 403 (2.7) |
| COPD | 2023 (13.7) | 115 (0.8) |
| Chronic kidney dysfunction | 2743 (18.5) | 113 (0.8) |
| ICD therapy | 3760 (25.4) | 125 (0.8) |
| Left Ventricular Ejection Fraction |  |  |
| <40 | 7981 (53.9) | 1528 (10.3) |
| 40-49 | 2731 (18.4) |  |
| >=50 | 2579 (17.4) |  |
| NYHA III/IV | 3936 (26.6) | 123 (0.8) |
| Either pulmonary rales, hepatomegaly, peripheral edema | 4436 (29.9) | 417 (2.8) |
| Third heart sound | 781 (5.3) | 467 (3.2) |
| Moderate severe Aortic Stenosis | 469 (3.2) | 2555 (17.2) |
| ACE | 9879 (66.7) | 91 (0.6) |
| ARB | 3877 (26.2) | 83 (0.6) |
| ACE and/or ARB | 13007 (87.8) | 91 (0.6) |
| Beta Blocker | 13172 (88.9) | 84 (0.6) |
| Mortality (Dead) | 772 (6.6) | 3122 (21.1) |
| Follow-up time, days (standard deviation) | 343.2 (58.2) | 3122 (21.1) |
| Follow up time among Survivors | 354.4 (31.7) |  |
| Follow up time among Deceased | 184.7 (101.3) |  |
| **Hospitals' characteristics (N=322)** | **Observed data** | **Missing** |
| Catheterization laboratories | 174 (54.0) | 98 (30.4) |
| Electrophysiological laboratories | 137 (42.6) | 107 (33.2) |
| Cardiology service: Echocardiography | 116 (36.0) | 81 (25.2) |
| Cardiology service: Angiography/PCI | 81 (25.2) | 141 (44) |
| Heart failure unit/clinic | 152 (47.2) | 87 (27.0) |
| Heart transplant | 40 (12.4) | 170 (52.8) |
| **Countries' characteristics (N=32)** | **Observed data** | **Missing N(%)** |
| GDP per capita (PPP) (international $) | 30 648.1 | (13 445.02) |
| Gini index | 33.2 | (4.5) |
| Health Expenditure per capita (PPP) | 1 926 | (1 658.72) |
| Total Health Expenditure on GDP (%) | 8.3 | (2.08) |
| LE | 78.1 | (3.79) |
| N deaths per cardiovascular diseases (per million people) | 6 134 | (3 111.33) |

**S1.1 Full names of the Institutions of each National Coordinator in which the protocol was approved**

**Austria**: Medical University, Graz, Austria

**Belarus**: Republican Scientific and Practical Centre of Cardiology, Minsk, Belarus

**Bosnia & Herzegovina**: Clinical Hospital Mostar, Mostar, Bosnia and Herzegovina

**Bulgaria**: University Hospital Lozenets, Sofia, Bulgaria

**Czech Republic**: Faculty General Hospital, Charles University, Prague, Czech Republic

**Cyprus**: Nicosia General Hospital, Nicosia, Cyprus

**Denmark**: Bispebjerg University Hospital, Copenhagen, Denmark

**Egypt**: Alexandria University, Alexandria, Egypt

**Estonia**: Faculty of Medicine, University of Tartu, Estonia

**France**: Assistance Publique Hôpitaux Paris, Hôpital Lariboisière, Université Paris Diderot, Inserm 942, Paris, France

**Greece**: Athens University Medical School, Athens, Greece

**Hungary**: Semmelweis University, Budapest, Hungary

**Israel**: Poriya Medical Centre and Faculty of Medicine Bar Ilan University, Israel

**Italy**: Department of Medical and Surgical Specialties, Radiological Sciences and Public Health, University of Brescia, Brescia, Italy

**Lithuania**: Lithuanian University of Health Sciences, Kaunas, Lithuania

**Poland**: Medical University of Lodz, Lodz, Poland

**Portugal**: S Francisco Xavier Hospital/CHLO NOVA Medical School, Faculdade de Ciências Medicas, Universidade Nova de Lisboa, Portugal

**Serbia**: University of Belgrade, School of Medicine, Serbia

**Slovakia**: National Cardiovascular Institute, Bratislava, Slovakia

**Slovenia**: General Hospital Celje, Celje, Slovenia

**Spain**: Complexo Hospitalario Universitario A Coruna, CHUAC, La Coruna, Spain

**Turkey**: Yüksek Ihtisas Hospital, Cardiology Clinic, Ankara, Turkey

**S1.2 Multiple Imputation Approach**

The implementation of the multiple imputation approach was developed as a systematic stepwise data-driven approach to identifying predictors of non-response.

The variables used to do multiple imputation comprise (1) those that are part of the model (i.e. exposures, outcome and controls) and (2) auxiliary variables, i.e. variables whose sole purpose is to improve the performance of the missing data methods, even when they are not relevant to the hypotheses of scientific interest (Collins et al, 2001^[[1]](#footnote-1)^). In the context of survival data, it is important to identify what to include in the imputation model as survival outcome. We follow the approach of White and Royston (2009)^[[2]](#footnote-2)^ as presented by Cleves et al. (2016)^[[3]](#footnote-3)^, and include the failure indicator (mortality) and the cumulative baseline hazard estimated using the Nelson-Aalen estimator.

Among auxiliary variables, we include (i) variables measured at baseline that have complete records and have been found to be strong predictors of missingness. This corresponded to the variable “home situation” (whether patient lives alone, with family/partner, in a nursing home, or other); (ii) a key predictor of the outcome (which is not necessarily a confounder) that we identified with “number of comorbidities” measured as the presence or absence of various conditions assessed at baseline; (iii) auxiliary variables that are associated with the missingness. The predictors of response were identified by a data-driven methods starting from univariate correlation with all variables included in the baseline survey (patient’s characteristics, medication and outpatient visit) that have less than 40% missing values; among these variables we selected those significantly associated with non-response and then run multivariate analysis. Finally, from multivariate analysis, we selected the strongest predictors of non-response. These are use of spirometry and use of nitrates during outpatient visits.

All selected variables were imputed through chain equations and imputing 20 datasets.

**S1.3 Discrete-time survival analysis**

We considered the piecewise exponential (PWE) model as the main model and run a discrete time survival model as supplementary analysis. Compared to the PWE, in the discrete time survival model the time scale is also divided into intervals and the hazard function is assumed to be constant within each interval. However, differently from PWE, within an interval each subject’s duration of exposure is not taken into account and only the occurrence of the event is considered^[[4]](#footnote-4)^. To fit discrete survival model, we used a logit link function and included country and hospital random effects.

**S1 Fig.** Crude mortality rate per 100 person year with 95% confidence interval, by country, sorted from lowest to highest rate.


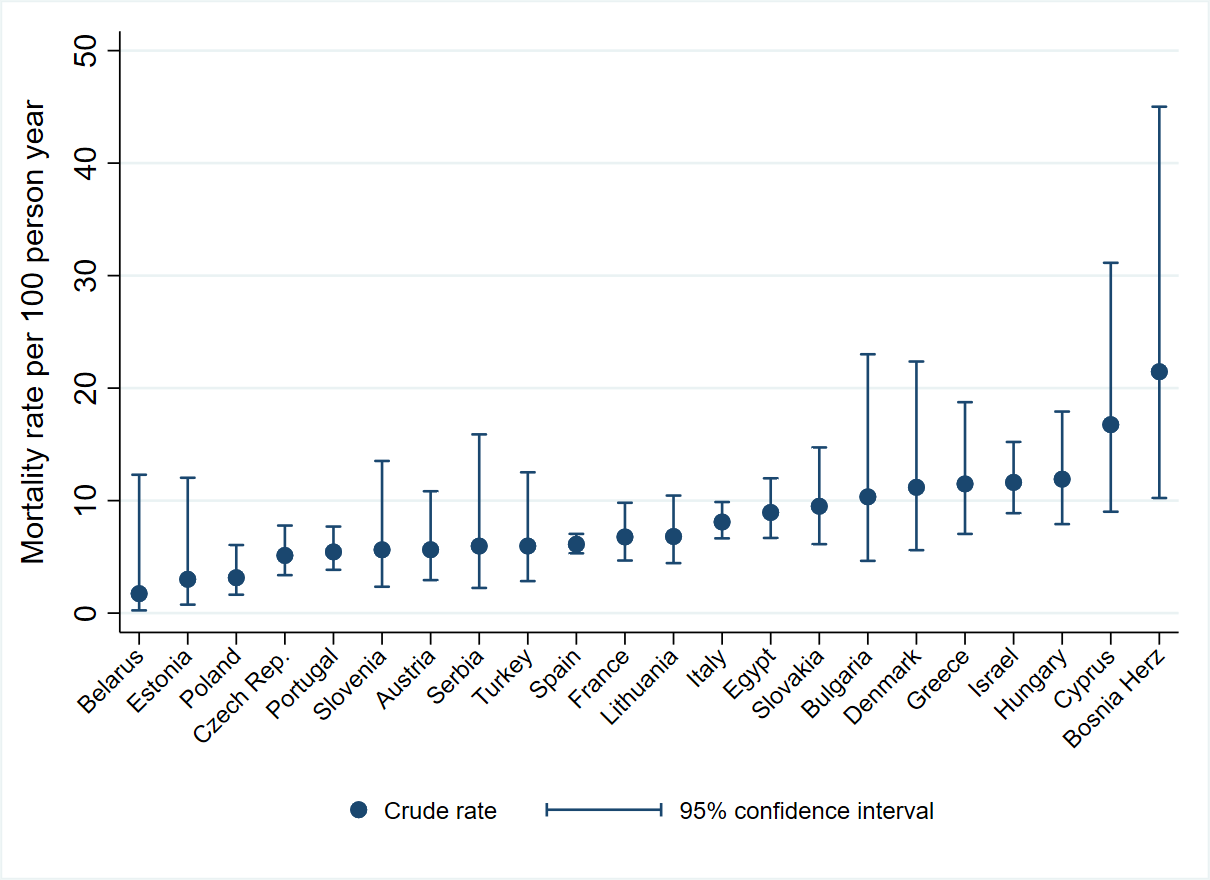


**S3 Table.** Baseline characteristics of the ESC member countries included in the study (N=22). Values are mean (standard deviation) unless stated otherwise.

| **Indicators** | **Mean (sd)** |
| --- | --- |
| GDP per capita at Purchasing Price Parity (in US $) | 31 140.4 (10 927.6) |
| Gini index | 33.29 (4.61) |
| Health Expenditure per capita at Purchasing Price Parity | 1926 (1 658.7) |
| Total Health Expenditure on GDP (in US$) | 8.32 (2.01) |
| Life Expectancy (years) | 78.69 (3.43) |
| N deaths per cardiovascular diseases (per million people per year) | 5625.6 (3 031.26) |

Data are available for all countries, hence missing values are not reported

**S4 Table.** PWE survival model using complete case analysis, N=5,773.

|  | Patient's characteristics | Patient + Hospital characteristics | Patient + Country characteristics | Patient + Hospital + Country characteristics |
| --- | --- | --- | --- | --- |
|  | IRR (95% CI) | IRR (95% CI) | IRR (95% CI) | IRR (95% CI) |
| ***Clinical variables*** |  |  |  |  |
| Males (vs females) | 1.36** (1.04 - 1.78) | 1.37** (1.04 - 1.79) | 1.34** (1.02 - 1.77) | 1.36** (1.03 - 1.78) |
| Age in years | 1.03*** (1.02 - 1.04) | 1.03*** (1.02 - 1.04) | 1.03*** (1.02 - 1.05) | 1.03*** (1.02 - 1.05) |
| BMI (Kg/m2) (20-24.9) vs BMI<20 | 0.43** (0.19 - 0.99) | 0.39** (0.169 - 0.90) | 0.40** (0.17 - 0.93) | 0.38** (0.17 - 0.89) |
| BMI (Kg/m2) (25-29.9) vs BMI<20 | 0.27*** (0.11 - 0.62) | 0.24*** (0.10 - 0.56) | 0.25*** (0.11 - 0.58) | 0.24*** (0.10 - 0.55) |
| BMI (Kg/m2) (30-34.9) vs BMI<20 | 0.26*** (0.11 - 0.62) | 0.23*** (0.097 - 0.55) | 0.24*** (0.10 - 0.57) | 0.22*** (0.09 - 0.54) |
| BMI (Kg/m2) (>=35) vs BMI<20 | 0.27*** (0.11 - 0.68) | 0.24*** (0.098 - 0.61) | 0.25*** (0.10 - 0.63) | 0.24*** (0.10 - 0.59) |
| Systolic blood pressure, mmHg | 0.92*** (0.90 - 0.95) | 0.92*** (0.90 - 0.95) | 0.92*** (0.90 - 0.95) | 0.92*** (0.90 - 0.95) |
| Ischemic etiology | 1.00 (0.79 - 1.26) | 0.99 (0.79 - 1.25) | 0.97 (0.77 - 1.23) | 0.98 (0.78 - 1.24) |
| Atrial Fibrillation history | 1.21* (0.97 - 1.51) | 1.22* (0.98 - 1.52) | 1.21* (0.968 - 1.51) | 1.22* (0.98 - 1.525) |
| Diabetes history | 1.48*** (1.18 - 1.85) | 1.49*** (1.19 - 1.87) | 1.48*** (1.18 - 1.85) | 1.49*** (1.19 - 1.86) |
| Peripheral artery disease | 1.67*** (1.27 - 2.20) | 1.59*** (1.20 - 2.11) | 1.69*** (1.28 - 2.22) | 1.59*** (1.20 - 2.11) |
| Chronic obstructive pulmonary disease | 1.10 (0.84 - 1.44) | 1.12 (0.86 - 1.47) | 1.12 (0.86 - 1.47) | 1.14 (0.87 - 1.49) |
| Chronic kidney dysfunction | 1.72*** (1.36 - 2.16) | 1.72*** (1.36 - 2.17) | 1.73*** (1.37 - 2.18) | 1.72*** (1.37 - 2.17) |
| Implantable cardioverter defibrillator therapy | 0.97 (0.75 - 1.24) | 1.01 (0.78 - 1.30) | 1.00 (0.78 - 1.29) | 1.00 (0.78 - 1.30) |
| New York Heart Association (NYHA) (III/IV vs I/II) | 2.10*** (1.66 - 2.67) | 2.10*** (1.66 - 2.67) | 2.10*** (1.65 - 2.67) | 2.12*** (1.67 - 2.69) |
| Peripheral oedema/ pulmonary rale | 1.84*** (1.45 - 2.35) | 1.78*** (1.40 - 2.28) | 1.76*** (1.38 - 2.25) | 1.73*** (1.36 - 2.22) |
| s3gallop | 1.34 (0.92 - 1.95) | 1.31 (0.91 - 1.91) | 1.26 (0.87 - 1.84) | 1.30 (0.89 - 1.89) |
| Medication Angiotensin-converting enzyme (ACE)/ Angiotensin receptor blocker (ARB) | 0.75** (0.57 - 1.00) | 0.75** (0.57 - 0.99) | 0.75** (0.56 - 0.99) | 0.76* (0.57 - 1.01) |
| Medication beta blocker | 0.77 (0.56 - 1.05) | 0.79 (0.58 - 1.09) | 0.80 (0.58 - 1.10) | 0.82 (0.60 - 1.13) |
| Left ventricular ejection fraction (EF) 40-49 (vs EF<40) | 0.92 (0.68 - 1.25) | 0.91 (0.67 - 1.24) | 0.91 (0.67 - 1.24) | 0.90 (0.66 - 1.22) |
| EF>=50 (vs EF<40) | 0.77 (0.55 - 1.07) | 0.75* (0.54 - 1.05) | 0.78 (0.56 - 1.08) | 0.75* (0.54 - 1.05) |
| Interval 90-180 days | 1.16 (0.85 - 1.577) | 1.16 (0.85 - 1.58) | 1.16 (0.85 - 1.58) | 1.16 (0.85 - 1.58) |
| 180-270 days | 1.45** (1.08 - 1.95) | 1.45** (1.08 - 1.95) | 1.45** (1.08 - 1.95) | 1.45** (1.08 - 1.95) |
| 270-365 days | 1.45** (1.07 - 1.97) | 1.45** (1.07 - 1.97) | 1.45** (1.07 - 1.97) | 1.45** (1.07 - 1.97) |
| ***Hospital’s characteristics*** |  |  |  |  |
| Catheterisation lab |  | 1.14 (0.52 - 2.45) |  | 0.72 (0.29 - 1.79) |
| Electrophysiological lab |  | 0.72 (0.448 - 1.16) |  | 0.70 (0.43 - 1.15) |
| Cardiology Echocardiography service 24 hrs on site (vs Regular hours) |  | 0.88 (0.64 - 1.21) |  | 0.94 (0.68 - 1.31) |
| Cardiology Angiography/PCI service 24 hrs on site (vs Regular hours) |  | 1.29 (0.91 - 1.84) |  | 1.27 (0.89 - 1.81) |
| Heart failure unit |  | 0.50*** (0.32 - 0.79) |  | 0.51*** (0.31 - 0.83) |
| Heart transplantation |  | 1.00 (0.71 - 1.40) |  | 1.10 (0.78 - 1.56) |
| ***Country’s characteristics*** |  |  |  |  |
| Gross Domestic Product (GDP) (in thousands $) |  |  | 0.92 (0.83 - 1.03) | 0.92 (0.82 - 1.05) |
| Life Expectancy at birth (years) |  |  | 1.10 (0.77 - 1.55) | 1.37 (0.91 - 2.07) |
| Total health expenditure (% of GDP) |  |  | 0.92 (0.74 - 1.16) | 0.80 (0.61 - 1.05) |
| Gini Index |  |  | 0.97 (0.88 - 1.07) | 1.01 (0.90 - 1.12) |
| Health expenditure per capita (PPP) (in thousands $) |  |  | 1.28 (0.61 - 2.68) | 1.57 (0.63 - 3.91) |
| Cardiovascular disease deaths per million inhabitants (in thousands per year) |  |  | 0.99 (0.70 - 1.42) | 1.27 (0.84 - 1.93) |
| Health system Bismarck (vs Beveridge) |  |  | 0.88 (0.30 - 2.61) | 0.65 (0.19 - 2.23) |
| Health system Semashko (vs Beveridge) |  |  | 1.06 (0.27 - 4.20) | 0.80 (0.16 - 3.99) |
| **Random Effects** |  |  |  |  |
| Country variance | 4.11E-15 (.-.) | 0.0542 (8.86E-03 - 0.3676) | 7.42e-21 (.-.) | 0.0384 (0.0060 - 0.2640) |
| Hospital variance | 0.0628 (0.0387-0.3357) | 0.0357 (0.0004 - 0.8352) | 0.0478 (0.0204 - 0.2631) | 0.0298 (4.95E-06 - 12.6221) |
| Observations | 21,784 | 21,784 | 21,784 | 21,784 |
| Number of hospitals | 73 | 73 | 73 | 73 |
| Number of countries | 19 | 19 | 19 | 19 |

**IRR**=incidence rate ratio; 95% CI in parentheses; *** p<0.01, ** p<0.05, * p<0.1

**S5 Table.** Discrete-time survival model for predictors of all-cause of mortality in patients with chronic HF, odds ratios and 95% confidence intervals.

|  | Multiple Imputation | Complete Case Analysis |
| --- | --- | --- |
|  | OR (95% CI) | OR (95% CI) |
| ***Clinical variables*** |  |  |
| Males (vs females) | 1.23* (0.996 - 1.510) | 1.37** (1.039 - 1.813) |
| Age in years | 1.03*** (1.019 - 1.036) | 1.03*** (1.023 - 1.046) |
| BMI (Kg/m2) (20-24.9) vs BMI<20 | 0.63 (0.294 - 1.349) | 0.38** (0.158 - 0.918) |
| BMI (Kg/m2) (25-29.9) vs BMI<20 | 0.40** (0.183 - 0.851) | 0.23*** (0.095 - 0.557) |
| BMI (Kg/m2) (30-34.9) vs BMI<20 | 0.385** (0.176 - 0.842) | 0.22*** (0.089 - 0.545) |
| BMI (Kg/m2) (>=35) vs BMI<20 | 0.381** (0.167 - 0.865) | 0.23*** (0.088 - 0.593) |
| Systolic blood pressure, mmHg | 0.924*** (0.903 - 0.945) | 0.92*** (0.894 - 0.950) |
| Ischemic etiology | 1.113 (0.922 - 1.342) | 0.98 (0.770 - 1.242) |
| Atrial Fibrillation history | 1.088 (0.913 - 1.298) | 1.23* (0.980 - 1.545) |
| Diabetes history | 1.421*** (1.184 - 1.704) | 1.51*** (1.196 - 1.894) |
| Peripheral artery disease | 1.613*** (1.299 - 2.002) | 1.61*** (1.203 - 2.155) |
| Chronic obstructive pulmonary disease | 1.181 (0.955 - 1.460) | 1.14 (0.864 - 1.506) |
| Chronic kidney dysfunction | 1.872*** (1.552 - 2.257) | 1.74*** (1.370 - 2.204) |
| Implantable cardioverter defibrillator therapy | 0.967 (0.784 - 1.192) | 0.99 (0.762 - 1.289) |
| New York Heart Association (NYHA) (III/IV vs I/II) | 2.052*** (1.700 - 2.477) | 2.17*** (1.704 - 2.766) |
| Peripheral oedema/ pulmonary rale | 1.872*** (1.546 - 2.267) | 1.76*** (1.370 - 2.265) |
| s3gallop | 1.072 (0.770 - 1.494) | 1.30 (0.884 - 1.914) |
| Medication Angiotensin-converting enzyme (ACE)/ Angiotensin receptor blocker (ARB) | 0.72*** (0.577 - 0.901) | 0.76* (0.563 - 1.012) |
| Medication beta blocker | 0.85 (0.657 - 1.090) | 0.82 (0.589 - 1.139) |
| Left ventricular ejection fraction (EF) 40-49 (vs EF<40) | 0.81* (0.626 - 1.036) | 0.88 (0.642 - 1.209) |
| EF>=50 (vs EF<40) | 0.78* (0.600 - 1.022) | 0.74* (0.523 - 1.034) |
| Interval 90-180 days | 1.16 (0.916 - 1.463) | 1.16 (0.849 - 1.596) |
| 180-270 days | 1.22 (0.961 - 1.536) | 1.45** (1.066 - 1.962) |
| 270-365 days | 1.25* (0.982 - 1.579) | 1.44** (1.052 - 1.958) |
| ***Hospital’s characteristics*** |  |  |
| Catheterisation lab | 1.30 (0.844 - 2.004) | 0.68 (0.266 - 1.716) |
| Electrophysiological lab | 0.89 (0.607 - 1.311) | 0.70 (0.422 - 1.146) |
| Cardiology Echocardiography service 24 hrs on site (vs Regular hours) | 0.99 (0.743 - 1.321) | 0.96 (0.687 - 1.335) |
| Cardiology Angiography/PCI service 24 hrs on site (vs Regular hours) | 0.94 (0.693 - 1.270) | 1.28 (0.890 - 1.843) |
| Heart failure unit | 0.51*** (0.360 - 0.721) | 0.49*** (0.295 - 0.810) |
| Heart transplantation | 0.91 (0.672 - 1.222) | 1.15 (0.804 - 1.634) |
| ***Country’s characteristics*** |  |  |
| Gross Domestic Product (GDP) (in thousands $) | 1.00 (0.942 - 1.069) | 0.92 (0.815 - 1.045) |
| Life Expectancy at birth (years) | 0.91 (0.757 - 1.097) | 1.38 (0.904 - 2.109) |
| Total health expenditure (% of GDP) | 1.06 (0.909 - 1.240) | 0.79* (0.603 - 1.039) |
| Gini Index | 9.29 (0.003 - 34,490.033) | 2.42 (0.000 - 181,690.903) |
| Health expenditure per capita (PPP) (in thousands $) | 1.15 (0.745 - 1.767) | 1.60 (0.629 - 4.047) |
| Cardiovascular disease deaths per million inhabitants (in thousands per year) | 1.02 (0.824 - 1.264) | 1.28 (0.833 - 1.958) |
| Health system Bismarck (vs Beveridge) | 1.22 (0.566 - 2.631) | 0.63 (0.180 - 2.228) |
| Health system Semashko (vs Beveridge) | 0.98 (0.396 - 2.438) | 0.78 (0.152 - 4.017) |
| **Random Effects** |  |  |
| Country variance (s.e.) ^§^ (95% CI) | 3.29E-06 (0.056) (0 - .) | 0.0403 (0.006 - 0.2745) |
| Hospital variance (95% CI) | 0.3032 (0.1751-0.5248) | 0.0304 (4.85E-07 - 81.835) |
| Observations | 35,812 | 21,784 |
| Number of hospitals | 142 | 73 |
| Number of countries | 22 | 19 |

**OR**=odds ratio95% CI in parentheses; *** p<0.01, ** p<0.05, * p<0.1

^§^ s.e. reported given 95% CI was not computable.

**S6 Table.** PWE survival model dropping observations from hospitals with less than or equal to 10 patients.

|  | Multiple Imputation | Complete Case Analysis |
| --- | --- | --- |
|  | IRR (95% CI) | IRR (95% CI) |
| ***Clinical variables*** |  |  |
| Males (vs females) | 1.20* (0.980 - 1.470) | 1.34** (1.019 - 1.766) |
| Age in years | 1.03*** (1.021 - 1.038) | 1.03*** (1.022 - 1.045) |
| BMI (Kg/m2) (20-24.9) vs BMI<20 | 0.66 (0.322 - 1.363) | 0.37** (0.160 - 0.863) |
| BMI (Kg/m2) (25-29.9) vs BMI<20 | 0.42** (0.203 - 0.869) | 0.24*** (0.101 - 0.550) |
| BMI (Kg/m2) (30-34.9) vs BMI<20 | 0.42** (0.198 - 0.877) | 0.23*** (0.095 - 0.543) |
| BMI (Kg/m2) (>=35) vs BMI<20 | 0.43** (0.196 - 0.931) | 0.24*** (0.095 - 0.596) |
| Systolic blood pressure, mmHg | 0.93*** (0.905 - 0.947) | 0.92*** (0.894 - 0.949) |
| Ischemic etiology | 1.10 (0.915 - 1.319) | 0.98 (0.778 - 1.244) |
| Atrial Fibrillation history | 1.11 (0.939 - 1.324) | 1.25* (0.996 - 1.561) |
| Diabetes history | 1.39*** (1.161 - 1.655) | 1.50*** (1.198 - 1.884) |
| Peripheral artery disease | 1.57*** (1.273 - 1.939) | 1.63*** (1.228 - 2.168) |
| Chronic obstructive pulmonary disease | 1.20* (0.975 - 1.469) | 1.11 (0.844 - 1.458) |
| Chronic kidney dysfunction | 1.77*** (1.477 - 2.128) | 1.68*** (1.331 - 2.125) |
| Implantable cardioverter defibrillator therapy | 1.00 (0.812 - 1.223) | 1.00 (0.769 - 1.289) |
| New York Heart Association (NYHA) (III/IV vs I/II) | 1.95*** (1.625 - 2.350) | 2.14*** (1.686 - 2.722) |
| Peripheral oedema/ pulmonary rale | 1.84*** (1.524 - 2.219) | 1.76*** (1.371 - 2.254) |
| s3gallop | 1.13 (0.825 - 1.546) | 1.31 (0.897 - 1.903) |
| Medication Angiotensin-converting enzyme (ACE)/ Angiotensin receptor blocker (ARB) | 0.70*** (0.563 - 0.866) | 0.77* (0.577 - 1.022) |
| Medication beta blocker | 0.83 (0.652 - 1.058) | 0.82 (0.594 - 1.134) |
| Left ventricular ejection fraction (EF) 40-49 (vs EF<40) | 0.85 (0.658 - 1.098) | 0.90 (0.662 - 1.230) |
| EF>=50 (vs EF<40) | 0.75** (0.577 - 0.983) | 0.73* (0.518 - 1.020) |
| Interval 90-180 days | 1.19 (0.946 - 1.497) | 1.19 (0.872 - 1.623) |
| 180-270 days | 1.26** (1.003 - 1.588) | 1.46** (1.077 - 1.967) |
| 270-365 days | 1.33** (1.056 - 1.679) | 1.47** (1.085 - 2.001) |
| ***Hospital’s characteristics*** |  |  |
| Catheterisation lab | 1.19 (0.768 - 1.848) | 0.74 (0.302 - 1.821) |
| Electrophysiological lab | 0.95 (0.643 - 1.407) | 0.69 (0.416 - 1.129) |
| Cardiology Echocardiography service 24 hrs on site (vs Regular hours) | 1.06 (0.786 - 1.433) | 0.97 (0.688 - 1.354) |
| Cardiology Angiography/PCI service 24 hrs on site (vs Regular hours) | 0.89 (0.656 - 1.220) | 1.26 (0.866 - 1.836) |
| Heart failure unit | 0.68** (0.489 - 0.954) | 0.52** (0.311 - 0.873) |
| Heart transplantation | 0.94 (0.681 - 1.301) | 1.17 (0.831 - 1.659) |
| ***Country’s characteristics*** |  |  |
| Gross Domestic Product (GDP) (in thousands $) | 0.97 (0.908 - 1.034) | 0.92 (0.809 - 1.040) |
| Life Expectancy at birth (years) | 0.89 (0.735 - 1.086) | 1.34 (0.890 - 2.011) |
| Total health expenditure (% of GDP) | 1.00 (0.840 - 1.196) | 0.80 (0.605 - 1.059) |
| Gini Index | 1.34 (0.000 - 8,799.637) | 1.01 (0.902 - 1.126) |
| Health expenditure per capita (PPP) (in thousands $) | 1.31 (0.845 - 2.037) | 1.61 (0.626 - 4.147) |
| Cardiovascular disease deaths per million inhabitants (in thousands per year) | 0.91 (0.713 - 1.150) | 1.22 (0.799 - 1.865) |
| Health system Bismarck (vs Beveridge) | 1.17 (0.513 - 2.665) | 0.63 (0.185 - 2.180) |
| Health system Semashko (vs Beveridge) | 0.89 (0.341 - 2.338) | 0.74 (0.146 - 3.793) |
| **Random Effects** |  |  |
| Country variance (s.e.) ^§^ (95% CI) | 2.42E-06 (0.172) (.-.) | 0.031 (0.036) (0.003-0.305) |
| Hospital variance (95% CI) | 0.355 (0.233-0.541) | (0.007 (1.21e-06-40.924) |
| Observations | 35142 | 21,570 |
| Number of hospitals | 105 | 73 |
| Number of countries | 22 | 17 |

**IRR**=incidence rate ratio; 95% CI in parentheses; *** p<0.01, ** p<0.05, * p<0.1

^§^ s.e. reported given 95% CI was not computable.

1. Collins, L. M., Schafer, J. L., & Kam, C. M. (2001). A comparison of inclusive and restrictive strategies in modern missing data procedures. *Psychological methods*, *6*(4), 330. [↑](#footnote-ref-1)
2. White, I. R., & Royston, P. (2009). Imputing missing covariate values for the Cox model. *Statistics in medicine*, *28*(15), 1982-1998. [↑](#footnote-ref-2)
3. Cleves, M., Gould, W., Gould, W. W., Gutierrez, R., & Marchenko, Y. (2008). *An introduction to survival analysis using Stata*. Stata press. [↑](#footnote-ref-3)
4. Austin, P. C. (2017). A tutorial on multilevel survival analysis: methods, models and applications. *International Statistical Review, 85*(2), 185-203. [↑](#footnote-ref-4)
