# Supplementary material for: Patient, hospital and country-level risk factors of all-cause mortality among patients with chronic heart failure: Prospective international cohort study
Source: PLoS One. 2021 May 10;16(5):e0250931. doi: 10.1371/journal.pone.0250931 (PMC8109791; doi:10.1371/journal.pone.0250931)
Supplement: S1 File — (PDF) [file pone.0250931.s002.pdf]

EURObservational Research Programme

Site:		Patient ID number:	

Heart Failure Long-Term Registry
Patient Characteristics

Inclusion criteria:

- All outpatients with HF seen at the clinics and those admitted for acute, pre-existing or new onset HF to participating centres during the enrolment period (1 day per week).
- Chronic Heart Failure: every outpatient with chronic HF diagnosed according to the clinical judgment of the responsible cardiologist of the participating centres.
- Acute Heart Failure: patients admitted to hospital for acute HF for whom an IV therapy for HF (inotropes, vasodilators or diuretics) is needed.

Exclusion criteria:

- There are no specific exclusion criteria, with the exception of age that should be higher than 18 years.

1.1 Basic Information

Date of visit/hospitalisation: |__/__/____| dd/mm/yyyy

Consent : ☐ No ☐ Yes If No, *skip to section 6.1 CRF Completed*

Type of patient (1): ☐ Outpatient ☐ Hospital

Date of birth (2): |__/____| mm/yyyy

Sex: ☐ Male ☐ Female

Height: |_____| cm

Weight: |_____| kg

BMI: *Automatic calculation*

Ethnic origin: ☐ Caucasian ☐ Black ☐ Asian ☐ Other

Home situation: ☐ Home alone ☐ Home with family ☐ Nursing home ☐ Other

HF history:
☐ No
☐ Yes, without previous hospitalisation
☐ Yes, with previous hospitalisation

If Yes, without previous hospitalisation:

☐ < 6 months

☐ 6-12 months

☐ > 12 months

If Yes, with previous hospitalisation, Date of last Hospitalisation: |__/__/____| dd/mm/yyyy
Days hospitalised prior year: |_____| days

Heart rate: |_____| beats/min

Blood pressure (Systolic/Diastolic):|_____| / |_____| mmHg

Primary Etiology[3]:
- ☐ Ischemic Heart Disease documented by coronary angiography
- ☐ Ischemic Heart Disease not documented by coronary angiography
- ☐ Hypertension
- ☐ Dilated Cardiomyopathy
- ☐ Valve disease
- ☐ Tachicardia related cardiomyopathy
- ☐ HFPEF Syndrome
- ☐ Other If Other, please specify: |________________________________|

If Valve disease, please specify:

Mitral:	☐ No	☐ Yes	Aortic:	☐ No	☐ Yes
Tricuspid:	☐ No	☐ Yes	Pulmonary:	☐ No	☐ Yes

Clinical trial[4]: ☐ No ☐ Yes

1.2 Clinical History

Risk Factors

Smoking status(5): ☐ Never ☐ Current ☐ Former

Atrial fibrillation: ☐ No ☐ Permanent ☐ Persistent ☐ Paroxysmal

Diabetes: ☐ No ☐ Yes ☐ Newly diagnosed

If Yes or Newly diagnosed, details: ☐ Dietary control
☐ Oral medication
☐ Insulin
☐ Oral and insulin

Alcohol: ☐ Never ☐ Former ☐ Yes sometimes ☐ Yes daily

Physical activities: ☐ None ☐ Moderate ☐ Intensive

Previous and current condition

MI/Angina:	☐ No ☐ Yes	CABG:	☐ No ☐ Yes
PCI:	☐ No ☐ Yes	Stroke/TIA:	☐ No ☐ Yes
Peripheral vascular disease:	☐ No ☐ Yes	Valvular surgery:	☐ No ☐ Yes
Hypertension Treatment:(6)	☐ No ☐ Yes	VTE:	☐ No ☐ Yes
COPD (Chronic obstructive pulmonary disease):	☐ No ☐ Yes	Chronic kidney dysfunction:(7)	☐ No ☐ Yes
Current malignant (cancer) disease:	☐ No ☐ Yes	Hepatic dysfunction:	☐ No ☐ Yes
Depression:	☐ No ☐ Yes	Parkinson:	☐ No ☐ Yes
Rheumatoid arthritis:	☐ No ☐ Yes		

Sleep apnea: ☐ No ☐ Yes If Yes, Sleep apnea tested: ☐ No ☐ Yes
Sleep apnea treated: ☐ No ☐ Yes

Spirometry: ☐ No ☐ Yes If Yes, ☐ Normal ☐ Abnormal

If Abnormal, ☐ Gold 1 ☐ Gold 2 ☐ Gold 3 ☐ Gold 4

Forced Vital Capacity (FVC): |_____| L
Forced Expiratory Volume (FEV1): |_____| L
FEV/FVC: (automatic calculation)

Device therapy: ☐ No
☐ Pacemaker
☐ CRT-P
☐ CRT-D
☐ ICD for primary prevention
☐ ICD for secondary prevention

Thyroid dysfunction: ☐ No ☐ Hypothyroidism ☐ Hyperthyroidism

Hepatitis(8): ☐ No ☐ A ☐ B ☐ C

Last known Ejection Fraction available: ☐ No ☐ Yes

If Yes, |_____| % Method: ☐ Echocardiogram ☐ Angiography ☐ Scintigraphy ☐ CMR

Heart Failure Long-Term Registry
Outpatient visit

(To be completed only if type of patient = Outpatient)

2.1 Physical Signs

NYHA class: ☐ NYHA I ☐ NYHA II ☐ NYHA III ☐ NYHA IV

Pulmonary rales:	☐ No ☐ Yes	S3 gallop:	☐ No ☐ Yes
JVP(>6cm):	☐ No ☐ Yes	Peripheral hypoperfusion:	☐ No ☐ Yes
Pleural effusion:	☐ No ☐ Yes	Cold:	☐ No ☐ Yes
Hepatomegaly:	☐ No ☐ Yes	Mitral regurgitation:	☐ No ☐ Yes
Peripheral oedema:	☐ No ☐ Yes	Aortic stenosis:	☐ No ☐ Yes

2.2 Chemistry at Outpatient Visit (most recent)

White blood cells:	|_______|	☐ Cells/microL<br>☐ Giga/L	Total cholesterol:	|_______|	☐ mg/dL<br>☐ mmol/L
Hemoglobin:	|_______|	☐ g/dL<br>☐ mmol/L<br>☐ g/L	Fasting glucose:	|_______|	☐ mg/dL<br>☐ mmol/L
S-creatinine:	|_______|	☐ mg/dL<br>☐ µmol/L	HbA1c:	|_______|	☐ %<br>☐ mmol/mol
Nitrogen measured by:	☐ BUN	☐ Urea	BNP:	|_______|	☐ pg/mL<br>☐ pmol/L
If BUN(9):	|_______|	☐ mg/dL<br>☐ mmol/L	NT-proBNP:	|_______|	☐ pg/mL<br>☐ pmol/L
If urea:	|_______|	☐ mg/dL<br>☐ mmol/L	Sodium:	|_______|	mEq/L or mmol/L
Uric acid:	|_______|	☐ mg/dL<br>☐ µmol/L	Potassium:	|_______|	mEq/L or mmol/L
Proteinuria:	☐ No	☐ Yes	Bilirubin:	|_______|	☐ mg/dL<br>☐ µmol/L
TSH:	|_______|	mIU/L	HIV infection:	☐ No	☐ Yes
Troponin I or T:	|_______|	ng/mL or pg/mL	Hs-CRP:	|_______|	mg/L
Hs-Troponin I or T:	|_______|	ng/mL or pg/mL			

2.3 Investigations/Procedures

(In general the most recent results at the time of the visit. If in doubt select 'Not performed')

ECG: ☐ Performed ☐ Not performed If performed, date: |__/__/____| dd/mm/yyyy

Rhythm: ☐ Sinus ☐ Atrial fibrillation/flutter ☐ Paced ☐ Other
Heart rate: |________| beats/min
QRS-duration: |________| ms
QT-duration: |________| ms
LBBB: ☐ No ☐ Yes
LVH: ☐ No ☐ Yes
Pathological Q-wave: ☐ No ☐ Yes

QTc-length automatic calculation
Bazett
Fridericia

Chest X-ray: ☐ Performed ☐ Not performed Date: |__/__/____| dd/mm/yyyy

Normal? ☐ No ☐ Yes
If No, please specify:

Cardiac enlargement: ☐ No ☐ Yes	Pulmonary congestion: ☐ No ☐ Yes
Alveolar oedema: ☐ No ☐ Yes	Other abnormality: ☐ No ☐ Yes

If Other, please describe: |________________________________|

Echo-Doppler: ☐ Performed ☐ Not performed Date: |__/__/____| dd/mm/yyyy

EF: |________| %
LVEDD: |________| mm
LVH: ☐ No ☐ Yes
E/A: |________| ratio
Deceleration time: |________| ms
LA measurement: ☐ Volume ☐ Dimension ☐ Unknown
IF Volume, LA Volume: |________| ml
IF Dimension, LA Dimension: |________| cm
Restrictive/pseudonormal pattern: ☐ No ☐ Yes ☐ Not evaluated
Mitral regurgitation moderate-severe: ☐ No ☐ Yes
Aortic stenosis moderate-severe: ☐ No ☐ Yes
Aortic regurgitation moderate-severe: ☐ No ☐ Yes
Tricuspid regurgitation moderate-severe: ☐ No ☐ Yes

Exercise test: ☐ No ☐ Yes ☐ Patient cannot do test Date: |__/__/____| dd/mm/yyyy

Peak exercise, cycle ergometer: |________| watt
Peak exercise, treadmill: |________| metres
Peak VO2: |________| ml/kg/min
6 min walk test: |________| metres

Holter Monitoring: ☐ Performed ☐ Not performed Date: |__/__/____| dd/mm/yyyy

Mean HR: |________| beats/min
PVC hour: |________| complexes/24h
Unsustained VT: ☐ No ☐ Yes
Sustained VT: ☐ No ☐ Yes
Atrial fibrillation: ☐ No ☐ Yes

Coronary Angiography: ☐ Performed ☐ Not performed Date: |__/__/____| dd/mm/yyyy

Cardiac CT: ☐ Performed ☐ Not performed Date: |__/__/____| dd/mm/yyyy

PCI/CABG: ☐ Performed ☐ Not performed Date: |__/__/____| dd/mm/yyyy

EPS (Electrophysiological Study)**:** ☐ Performed ☐ Not performed Date: |__/__/____| dd/mm/yyyy

Inducible sustained VT/VF[10]: ☐ No ☐ Yes
Inducible atrial fibrillation: ☐ No ☐ Yes
Major conduction abnormalities: ☐ No ☐ Yes

Transcatheter Ablation: ☐ Performed ☐ Not performed Date: |__/__/____| dd/mm/yyyy

Atrial: ☐ No ☐ Yes
Ventricular: ☐ No ☐ Yes
Nodal: ☐ No ☐ Yes

Electric cardioversion: ☐ Performed ☐ Not performed Date: |__/__/____| dd/mm/yyyy

Atrial Fibrillation: ☐ No ☐ Yes
VT/VF: ☐ No ☐ Yes

Right Heart Catheterization: ☐ Performed ☐ Not performed Date: |__/__/____| dd/mm/yyyy

mPAP: |________| mmHg
Right atrial pressure: |________| mmHg
PCW: |________| mmHg
CI: |________| L/min/m²

Myocardial Scintigraphy: ☐ Performed ☐ Not performed Date: |__/__/____| dd/mm/yyyy

Resting ischemia : ☐ No ☐ Yes
Myocardial viability: ☐ No ☐ Yes

Endomyocardial Biopsy: ☐ Performed ☐ Not performed Date: |__/__/____| dd/mm/yyyy

IAPB: ☐ Performed ☐ Not performed Date: |__/__/____| dd/mm/yyyy

CRT implantation: ☐ Not indicated ☐ Indicated ☐ Already implanted

If indicated, treatment: ☐ Not planned ☐ Planned

If not planned, reason:
☐ Absence of clinical indication
☐ Cost issues
☐ Patient refusal
☐ Logistic issues
☐ Other

ICD implantation: ☐ Not indicated ☐ Indicated ☐ Already implanted

If indicated, treatment: ☐ Not planned ☐ Planned

If not planned, reason:
☐ Absence of clinical indication
☐ Cost issues
☐ Patient refusal
☐ Logistic issues
☐ Other

2.4 Scores

Was prognosis evaluated using a risk score? ☐ No ☐ Yes

If Yes,
☐ SEATLE
☐ CHARM
☐ GISSI-HF
☐ MAGGIC
☐ MECKI
☐ HF ACTIONS
☐ EMPHASIS
☐ OTHER

If Other, please specify: |________________________________|

References

- SEATLE : Wayne C; Levy et al – Circulation 2006, 113, 1424-1433
- CHARM : Stuart J. Pocock et al – European Heart Journal 2006, 27, 65-75
- GISSI-HF : Simona Barlera et al – Circulation Heart Failure 2013, 6, 31-39
- MAGGIC : Stuart J. Pocock et al – European Heart Journal 2012, 34, 1404-13
- MECKI : Piergiuseppe Agostini et al – Interventional Journal of Cardiology, 2012, 06-113
- HF ACTION: Christopher M. O’Connor et al - Circulation Heart Failure 2012, 5, 63-71
- EMPHASIS : Timothy J. Collier et al - European Heart Journal 2013, 34, 2823-9

Heart Failure Long-Term Registry
Hospitalisation

(To be completed only if type of patient = Hospital Inpatient)

3.1 Hospitalised patients

First medical contact:
☐ Family doctor/GP ☐ Ambulance personnel
☐ Outpatient clinic ☐ Nurse
☐ Other

How did the patient arrive at the hospital?
☐ Own transport ☐ Ambulance and physician
☐ Ambulance and nurse ☐ Other

Where was the patient first admitted?
☐ Emergency room ☐ Medical ward
☐ Heart Failure facilities ☐ Cardiac/Coronary ICU
☐ Cardiac ward ☐ General/Medical/Surgical ICU
☐ Other

HF status: ☐ New onset ☐ Worsening

Reason for hospitalisation, precipitating factors:

Heart Failure:	☐ No	☐ Yes	ACS:	☐ No	☐ Yes
Myocardial ischemia:	☐ No	☐ Yes	Non compliance behavioural drugs:	☐ No	☐ Yes
Atrial fibrillation:	☐ No	☐ Yes	Ventricular arrhythmia:	☐ No	☐ Yes
Infection:	☐ No	☐ Yes	Uncontrolled hypertension:	☐ No	☐ Yes
Bradyarrhythmias:	☐ No	☐ Yes	Renal dysfunction:	☐ No	☐ Yes
Iatrogenic:	☐ No	☐ Yes	Anaemia:	☐ No	☐ Yes
Other:	☐ No	☐ Yes			

Hospital presentation, clinical profiles:
☐ Pulmonary oedema ☐ Hypertensive HF
☐ Cardiogenic shock ☐ Decompensated HF
☐ Right HF ☐ ACS/HF

Intropic support type:
☐ No ☐ Enoximone
☐ Dobutamine ☐ Levosimendan
☐ Dopamine ☐ Norepinephrine
☐ Milrinone ☐ Other

Hours of treatment: |________|

If Other, please specify: |____|

Nitrates IV: ☐ No ☐ Yes

Duration of Nitrate IV infusion: ☐ < 1 hr ☐ 1-3 hrs ☐ 3-6 hrs ☐ 6-12 hrs ☐ > 12 hrs

Reason for Nitrate IV infusion terminated:
☐ Clinically stabilised
☐ Low BP
☐ Headache
☐ Tachyphylaxis
☐ Other

Diuretics IV: ☐ No ☐ Yes

3.2 Physical Signs

NYHA class: ☐ NYHA II ☐ NYHA III ☐ NYHA IV

Pulmonary rales:	☐ No ☐ Yes	S3 gallop:	☐ No ☐ Yes
JVP(>6cm):	☐ No ☐ Yes	Peripheral hypoperfusion:	☐ No ☐ Yes
Pleural effusion:	☐ No ☐ Yes	Cold:	☐ No ☐ Yes
Hepatomegaly:	☐ No ☐ Yes	Mitral regurgitation:	☐ No ☐ Yes
Peripheral oedema:	☐ No ☐ Yes	Aortic stenosis:	☐ No ☐ Yes

3.3 Chemistry at Hospital Entry

White blood cells:	|_______|	☐ Cells/microL<br>☐ Giga/L	Total cholesterol:	|_______|	☐ mg/dL<br>☐ mmol/L
Hemoglobin:	|_______|	☐ g/dL<br>☐ mmol/L<br>☐ g/L	Fasting glucose:	|_______|	☐ mg/dL<br>☐ mmol/L
S-creatinine:	|_______|	☐ mg/dL<br>☐ µmol/L	HbA1c:	|_______|	☐ %<br>☐ mmol/mol
Nitrogen measured by:	☐ BUN	☐ Urea	BNP:	|_______|	☐ pg/mL<br>☐ pmol/L
If BUN[9]:	|_______|	☐ mg/dL<br>☐ mmol/L	NT-proBNP:	|_______|	☐ pg/mL<br>☐ pmol/L
If urea:	|_______|	☐ mg/dL<br>☐ mmol/L	Sodium:	|_______|	mEq/L or mmol/L
Uric acid:	|_______|	☐ mg/dL<br>☐ µmol/L	Potassium:	|_______|	mEq/L or mmol/L
Proteinuria:	☐ No	☐ Yes	Bilirubin:	|_______|	☐ mg/dL<br>☐ µmol/L
TSH:	|_______|	mIU/L	HIV infection:	☐ No	☐ Yes
Troponin I or T:	|_______|	ng/mL or pg/mL	Hs-CRP:	|_______|	mg/L
Hs-Troponin I or T:	|_______|	ng/mL or pg/mL			

Heart Failure Long-Term Registry
Medication: (Outpatients)

4.1 Medications and Doses

CV drugs: *Doses should be total given in one day.*

Drug type	Prior	During Outpatient Visit
ACE inhibitors	☐ No<br>☐ Ramipril<br>☐ Enalapril<br>☐ Perindopril<br>☐ Lisinopril<br>☐ Captopril<br>☐ Fosinopril<br>☐ Other<br>Daily dose: |______| mg	☐ No<br>☐ Ramipril<br>☐ Enalapril<br>☐ Perindopril<br>☐ Lisinopril<br>☐ Captopril<br>☐ Fosinopril<br>☐ Other<br>If No, ☐ Contraindicated ☐ Not tolerated ☐ Other<br>If Contraindicated, ☐ Bilateral renal stenosis ☐ Hyperkalemia ☐ Symptomatic hypotension ☐ Severe renal dysfunction ☐ Other<br>If Not tolerated, ☐ Cough ☐ Worsening renal function ☐ Symptomatic hypotension ☐ Hyperkalemia ☐ Angioedema ☐ Other<br>If Yes, Daily dose: |______| mg<br>Reason for target dose not reached ☐ Cough ☐ Worsening renal function ☐ Symptomatic hypotension ☐ Hyperkalemia ☐ Angioedema ☐ Still in uptitration ☐ Other
Angiotensin II receptor Blockers (ARB)	☐ No<br>☐ Candesartan<br>☐ Losartan<br>☐ Valsartan<br>☐ Other<br>Daily dose: |______| mg	☐ No<br>☐ Candesartan<br>☐ Losartan<br>☐ Valsartan<br>☐ Other<br>If No, ☐ Contraindicated ☐ Not tolerated ☐ Other<br>If Contraindicated, ☐ Bilateral renal stenosis ☐ Hyperkalemia ☐ Symptomatic hypotension ☐ Severe renal dysfunction ☐ Other<br>If Not tolerated, ☐ Worsening renal function ☐ Symptomatic hypotension ☐ Hyperkalemia ☐ Angioedema ☐ Other

		If Yes, Daily dose: |______| mg<br>Reason for target dose not reached: ☐ Worsening renal function ☐ Symptomatic hypotension ☐ Hyperkalemia ☐ Angioedema ☐ Still in uptitration ☐ Other
Sacubitril / Valsartan<br>(ARNI: Angiotensin Receptor Neprilysin Inhibitor)	☐ No<br>☐ Yes<br>Daily dose: |______| mg	☐ No<br>☐ Yes<br>If No, ☐ Contraindicated ☐ Not tolerated ☐ Other<br>If Yes, Daily dose: |______| mg
Beta blockers	☐ No<br>☐ Carvedilol<br>☐ Bisoprolol<br>☐ Metoprolol<br>☐ Nebivolol<br>☐ Other<br>Daily dose: |______| mg	☐ No<br>☐ Carvedilol<br>☐ Bisoprolol<br>☐ Metoprolol<br>☐ Nebivolol<br>☐ Other<br>If No, ☐ Contraindicated ☐ Not tolerated ☐ Other<br>If Contraindicated, ☐ Asthma ☐ Bradyarrhythmia ☐ PAD ☐ Symptomatic hypotension ☐ Other<br>If Not tolerated, ☐ Broncospasm ☐ Worsening PAD ☐ Worsening HF ☐ Bradyarrhythmia ☐ Sexual dysfunction ☐ Symptomatic hypotension ☐ Other<br>If Yes, Daily dose: |______| mg<br>Reason for target dose not reached: ☐ Broncospasm ☐ Worsening PAD ☐ Worsening HF ☐ Bradyarrhythmia ☐ Sexual dysfunction ☐ Symptomatic hypotension ☐ Still in uptitration ☐ Other
Mineralocorticoid receptor antagonists (Aldosterone antagonists)	☐ No<br>☐ Spironolactone<br>☐ Eplerenone<br>☐ Canrenone<br>☐ Other<br>Daily dose: |______| mg	☐ No<br>☐ Spironolactone<br>☐ Eplerenone<br>☐ Canrenone<br>☐ Other<br>If No, ☐ Contraindicated ☐ Not tolerated ☐ Other<br>If Contraindicated, ☐ Hyperkalemia ☐ Severe renal dysfunction ☐ Other<br>If Not tolerated, ☐ Hyperkalemia ☐ Worsening renal function

		☐ Gynecomastie<br>☐ Other<br>If Yes, Daily dose: |______| mg<br>Reason for target dose not reached: ☐ Hyperkalemia<br>☐ Worsening renal function<br>☐ Gynecomastie<br>☐ Still in uptitration<br>☐ Other
Diuretics: Oral	☐ No<br>☐ Bendrofluazide<br>☐ Chlorthalidone<br>☐ Hydrochlorothiazide<br>☐ Furosemide<br>☐ Indapamide<br>☐ Torasemide<br>☐ Bumetanide<br>☐ Other<br>Daily dose: |______| mg	☐ No<br>☐ Bendrofluazide<br>☐ Chlorthalidone<br>☐ Hydrochlorothiazide<br>☐ Furosemide<br>☐ Indapamide<br>☐ Torasemide<br>☐ Bumetanide<br>☐ Other<br>Daily dose: |______| mg
Diuretics: Oral (2nd medication)	☐ No<br>☐ Bendrofluazide<br>☐ Chlorthalidone<br>☐ Hydrochlorothiazide<br>☐ Furosemide<br>☐ Indapamide<br>☐ Torasemide<br>☐ Bumetanide<br>☐ Other<br>Daily dose: |______| mg	☐ No<br>☐ Bendrofluazide<br>☐ Chlorthalidone<br>☐ Hydrochlorothiazide<br>☐ Furosemide<br>☐ Indapamide<br>☐ Torasemide<br>☐ Bumetanide<br>☐ Other<br>Daily dose: |______| mg
Ivabradine	☐ No ☐ Yes<br>Daily dose: |______| mg	☐ No ☐ Yes<br>If Yes, Daily dose: |______| mg<br>If No, ☐ Atrial Fibrillation/Flutter<br>☐ EF > 35 %<br>☐ HR < 70 bpm<br>☐ Medication still not available<br>☐ Not tolerated<br>☐ Other<br>If Other: |__________________|
Digitalis	☐ No ☐ Yes	☐ No ☐ Yes
Statins	☐ No ☐ Yes	☐ No ☐ Yes
Antiplatelets	☐ No ☐ Yes	☐ No ☐ Yes
Anticoagulants	☐ No ☐ Yes	☐ No ☐ Yes
Amiodarone	☐ No ☐ Yes	☐ No ☐ Yes
Nitrates	☐ No ☐ Yes	☐ No ☐ Yes
Calcium channel blockers	☐ No ☐ Yes	☐ No ☐ Yes
Antiarrhythmics	☐ No ☐ Yes	☐ No ☐ Yes
Direct renin inhibitors	☐ No ☐ Yes	☐ No ☐ Yes

Non CV drugs:

Drug type	Prior	During Outpatient Visit
Treatment for COPD	☐ No<br>☐ Corticosteroids<br>☐ Beta2 agonists<br>☐ Anticholinergic agents<br>☐ Xanthine agents	☐ No<br>☐ Corticosteroids<br>☐ Beta2 agonists<br>☐ Anticholinergic agents<br>☐ Xanthine agents
Anti-diabetic drugs: Insulin	☐ No ☐ Yes	☐ No ☐ Yes
Anti-diabetic drugs: Oral	☐ Metformin<br>☐ Glitazones<br>☐ Incretins<br>☐ Sulphanylurea<br>☐ Other<br>☐ None	☐ Metformin<br>☐ Glitazones<br>☐ Incretins<br>☐ Sulphanylurea<br>☐ Other<br>☐ None
Allopurinol	☐ No ☐ Yes	☐ No ☐ Yes
NSAIDs:	☐ No ☐ Yes	☐ No ☐ Yes
Antidepressants:	☐ No ☐ Yes	☐ No ☐ Yes
Number of other non CV drugs:	|____|	|____|

Heart Failure Long-Term Registry
Medication: (Hospital Inpatients)

4.1 Medications and Doses

CV drugs: *Doses should be total given in one day.*

ACE inhibitors

Prior	During Hospitalisation	Discharge
☐ No<br>☐ Ramipril<br>☐ Enalapril<br>☐ Perindopril<br>☐ Lisinopril<br>☐ Captopril<br>☐ Fosinopril<br>☐ Other<br>Daily dose: |______| *mg*	☐ No<br>☐ Ramipril<br>☐ Enalapril<br>☐ Perindopril<br>☐ Lisinopril<br>☐ Captopril<br>☐ Fosinopril<br>☐ Other<br>Daily dose: |______| *mg*	☐ No<br>☐ Ramipril<br>☐ Enalapril<br>☐ Perindopril<br>☐ Lisinopril<br>☐ Captopril<br>☐ Fosinopril<br>☐ Other<br>If No, ☐ Contraindicated<br>☐ Not tolerated<br>☐ Other<br>If Contraindicated,<br>☐ Bilateral renal stenosis<br>☐ Hyperkalemia<br>☐ Symptomatic hypotension<br>☐ Severe renal dysfunction<br>☐ Other<br>If Not tolerated,<br>☐ Cough<br>☐ Worsening renal function<br>☐ Symptomatic hypotension<br>☐ Hyperkalemia<br>☐ Angioedema<br>☐ Other<br>If Yes, Daily dose: |______| *mg*<br>Reason for target dose not reached:<br>☐ Cough<br>☐ Worsening renal function<br>☐ Symptomatic hypotension<br>☐ Hyperkalemia<br>☐ Angioedema<br>☐ Still in uptitration<br>☐ Other

Angiotensin II Receptor Blockers (ARB)

Prior	During Hospitalisation	Discharge
☐ No<br>☐ Candesartan<br>☐ Losartan<br>☐ Valsartan<br>☐ Other<br>Daily dose: |______| *mg*	☐ No<br>☐ Candesartan<br>☐ Losartan<br>☐ Valsartan<br>☐ Other<br>Daily dose: |______| *mg*	☐ No<br>☐ Candesartan<br>☐ Losartan<br>☐ Valsartan<br>☐ Other<br><br>If No, ☐ Contraindicated<br>☐ Not tolerated<br>☐ Other<br><br>If Contraindicated,<br>☐ Bilateral renal stenosis<br>☐ Hyperkalemia<br>☐ Symptomatic hypotension<br>☐ Severe renal dysfunction<br>☐ Other<br>If Not tolerated,<br>☐ Worsening renal function<br>☐ Symptomatic hypotension<br>☐ Hyperkalemia<br>☐ Angioedema<br>☐ Other<br>If Yes, Daily dose: |______| *mg*<br>Reason for target dose not reached:<br>☐ Worsening renal function<br>☐ Symptomatic hypotension<br>☐ Hyperkalemia<br>☐ Angioedema<br>☐ Still in uptitration<br>☐ Other

Sacubitril / Valsartan ARNI: Angiotensin Receptor Neprilysin Inhibitor)

Prior	During Hospitalisation	Discharge
☐ No<br>☐ Yes<br>Daily dose: |______| mg	☐ No<br>☐ Yes<br>Daily dose: |______| mg	☐ No<br>☐ Yes<br><br>If No, ☐ Contraindicated ☐ Not tolerated ☐ Other<br>If Yes, Daily dose: |______| mg

Beta blockers

Prior	During Hospitalisation	Discharge
☐ No<br>☐ Carvedilol<br>☐ Bisoprolol<br>☐ Metoprolol<br>☐ Nebivolol<br>☐ Other<br>Daily dose: |______| *mg*	☐ No<br>☐ Carvedilol<br>☐ Bisoprolol<br>☐ Metoprolol<br>☐ Nebivolol<br>☐ Other<br>Daily dose: |______| *mg*	☐ No<br>☐ Carvedilol<br>☐ Bisoprolol<br>☐ Metoprolol<br>☐ Nebivolol<br>☐ Other<br>If No, ☐ Contraindicated<br>☐ Not tolerated<br>☐ Other<br>If Contraindicated,<br>☐ Asthma<br>☐ Bradyarrhythmia<br>☐ PAD<br>☐ Symptomatic hypotension<br>☐ Other<br>If Not tolerated,<br>☐ Broncospasm<br>☐ Worsening PAD<br>☐ Worsening HF<br>☐ Bradyarrhythmia<br>☐ Sexual dysfunction<br>☐ Symptomatic hypotension<br>☐ Other<br>If Yes, Daily dose: |______| *mg*<br>Reason for target dose not reached:<br>☐ Broncospasm<br>☐ Worsening PAD<br>☐ Worsening HF<br>☐ Bradyarrhythmia<br>☐ Sexual dysfunction<br>☐ Symptomatic hypotension<br>☐ Still in uptitration<br>☐ Other

Mineralocorticoid receptor antagonists

Prior	During Hospitalisation	Discharge
☐ No<br>☐ Spironolactone<br>☐ Eplerenone<br>☐ Canrenone<br>☐ Other<br>Daily dose: |______| *mg*	☐ No<br>☐ Spironolactone<br>☐ Eplerenone<br>☐ Canrenone<br>☐ Other<br>If No, ☐ Contraindicated<br>☐ Not tolerated<br>☐ Other<br>If Contraindicated,<br>☐ Hyperkalemia<br>☐ Severe renal dysfunction<br>☐ Other<br>If Not tolerated,<br>☐ Hyperkalemia<br>☐ Worsening renal function<br>☐ Gynecomastie<br>☐ Other<br>If Yes, Daily dose: |______| *mg*<br>Reason for target dose not reached:<br>☐ Hyperkalemia<br>☐ Worsening renal function<br>☐ Gynecomastie<br>☐ Still in uptitration<br>☐ Other	☐ No<br>☐ Spironolactone<br>☐ Eplerenone<br>☐ Canrenone<br>☐ Other<br>If No, ☐ Contraindicated<br>☐ Not tolerated<br>☐ Other<br>If Contraindicated,<br>☐ Hyperkalemia<br>☐ Severe renal dysfunction<br>☐ Other<br>If Not tolerated,<br>☐ Hyperkalemia<br>☐ Worsening renal function<br>☐ Gynecomastie<br>☐ Other<br>If Yes, Daily dose: |______| *mg*<br>Reason for target dose not reached:<br>☐ Hyperkalemia<br>☐ Worsening renal function<br>☐ Gynecomastie<br>☐ Still in uptitration<br>☐ Other

Diuretics oral

Prior	During Hospitalisation	Discharge
☐ No<br>☐ Bendrofluazide<br>☐ Chlorthalidone<br>☐ Hydrochlorotiazide<br>☐ Furosemide<br>☐ Indapamide<br>☐ Torasemide<br>☐ Bumetanide<br>☐ Other<br>Daily dose: |______| *mg*	☐ No<br>☐ Bendrofluazide<br>☐ Chlorthalidone<br>☐ Hydrochlorotiazide<br>☐ Furosemide<br>☐ Indapamide<br>☐ Torasemide<br>☐ Bumetanide<br>☐ Other<br>Daily dose: |______| *mg*	☐ No<br>☐ Bendrofluazide<br>☐ Chlorthalidone<br>☐ Hydrochlorotiazide<br>☐ Furosemide<br>☐ Indapamide<br>☐ Torasemide<br>☐ Bumetanide<br>☐ Other<br>Daily dose: |______| *mg*

Diuretics oral (2nd medication)

Prior	During Hospitalisation	Discharge
☐ No<br>☐ Bendrofluazide<br>☐ Chlorthalidone<br>☐ Hydrochlorotiazide<br>☐ Furosemide<br>☐ Indapamide<br>☐ Torasemide<br>☐ Bumetanide<br>☐ Other<br>Daily dose: |______| *mg*	☐ No<br>☐ Bendrofluazide<br>☐ Chlorthalidone<br>☐ Hydrochlorotiazide<br>☐ Furosemide<br>☐ Indapamide<br>☐ Torasemide<br>☐ Bumetanide<br>☐ Other<br>Daily dose: |______| *mg*	☐ No<br>☐ Bendrofluazide<br>☐ Chlorthalidone<br>☐ Hydrochlorotiazide<br>☐ Furosemide<br>☐ Indapamide<br>☐ Torasemide<br>☐ Bumetanide<br>☐ Other<br>Daily dose: |______| *mg*

Ivabradine

Prior	During Hospitalisation	Discharge
☐ No ☐ Yes<br>If Yes, Daily dose: |______| *mg*	☐ No ☐ Yes<br>If Yes, Daily dose: |______| *mg*<br>If No, ☐ Atrial Fibrillation/Flutter<br>☐ EF > 35 %<br>☐ HR < 70 bpm<br>☐ Medic. still not available<br>☐ Not tolerated<br>☐ Other<br>If Other: |_________________|	☐ No ☐ Yes<br>If Yes, Daily dose: |______| *mg*<br>If No, ☐ Atrial Fibrillation/Flutter<br>☐ EF > 35 %<br>☐ HR < 70 bpm<br>☐ Medic. still not available<br>☐ Not tolerated<br>☐ Other<br>If Other: |_________________|

Drug type	Prior	During Hospitalisation	Discharge
Digitalis	☐ No ☐ Yes	☐ No ☐ Yes	☐ No ☐ Yes
Statins	☐ No ☐ Yes	☐ No ☐ Yes	☐ No ☐ Yes
Antiplatelets	☐ No ☐ Yes	☐ No ☐ Yes	☐ No ☐ Yes
Anticoagulants	☐ No ☐ Yes	☐ No ☐ Yes	☐ No ☐ Yes
Amiodarone	☐ No ☐ Yes	☐ No ☐ Yes	☐ No ☐ Yes
Nitrates	☐ No ☐ Yes	☐ No ☐ Yes	☐ No ☐ Yes
Calcium channel blockers	☐ No ☐ Yes	☐ No ☐ Yes	☐ No ☐ Yes
Antiarrhythmics	☐ No ☐ Yes	☐ No ☐ Yes	☐ No ☐ Yes
Direct renin inhibitors	☐ No ☐ Yes	☐ No ☐ Yes	☐ No ☐ Yes

Non CV drugs:

Drug type	Prior	During Hospitalisation	Discharge
Treatment for COPD	☐ No<br>☐ Corticosteroids<br>☐ Beta2 agonists<br>☐ Anticholinergic agents<br>☐ Xanthine agents	☐ No<br>☐ Corticosteroids<br>☐ Beta2 agonists<br>☐ Anticholinergic agents<br>☐ Xanthine agents	☐ No<br>☐ Corticosteroids<br>☐ Beta2 agonists<br>☐ Anticholinergic agents<br>☐ Xanthine agents
Anti-diabetic drugs: Insulin	☐ No ☐ Yes	☐ No ☐ Yes	☐ No ☐ Yes
Anti-diabetic drugs: Oral	☐ Metformin<br>☐ Glitazones<br>☐ Incretins<br>☐ Sulphanylurea<br>☐ Other<br>☐ None	☐ Metformin<br>☐ Glitazones<br>☐ Incretins<br>☐ Sulphanylurea<br>☐ Other<br>☐ None	☐ Metformin<br>☐ Glitazones<br>☐ Incretins<br>☐ Sulphanylurea<br>☐ Other<br>☐ None
Allopurinol	☐ No ☐ Yes	☐ No ☐ Yes	☐ No ☐ Yes
NSAIDs:	☐ No ☐ Yes	☐ No ☐ Yes	☐ No ☐ Yes
Antidepressants:	☐ No ☐ Yes	☐ No ☐ Yes	☐ No ☐ Yes
Number of non CV drugs:	|_____|	|_____|	|_____|

Heart Failure Long-Term Registry
Discharge (hospitalised patients)

5.1 Discharge/Outcome

Vital status: ☐ Alive ☐ Dead   Date of discharge/death: |__/__/____| dd/mm/yyyy

If Dead,

Causes of death: ☐ Procedure related ☐ Non procedure related ☐ Unknown

Causes of death: ☐ Cardiac ☐ Vascular ☐ Non cardiovascular ☐ Unknown

If cardiac causes, Mode: ☐ Sudden ☐ Non sudden

Causes: ☐ AMI ☐ Heart Failure ☐ Arrhythmia ☐ Other

If other cardiac cause, please specify: |____________________________|

If vascular causes, Details:
- ☐ Ischemic stroke
- ☐ Hemorrhagic stroke
- ☐ Systemic hemorrhage
- ☐ Peripheral embolism
- ☐ Pulmonary embolism

Time in Intensive Cardiac Care Unit: |_____| days

5.2 Biometrics

If vital status = Alive,
Weight: |______| kg
Blood pressure (Systolic/Diastolic):|_____| / |_____| mmHg
Heart rate: |______| beats/min
NYHA class: ☐ NYHA I ☐ NYHA II ☐ NYHA III ☐ NYHA IV

5.3 Physical Signs

Pulmonary rales:	☐ No ☐ Yes	S3 gallop:	☐ No ☐ Yes
JVP(>6cm):	☐ No ☐ Yes	Peripheral hypoperfusion:	☐ No ☐ Yes
Pleural effusion:	☐ No ☐ Yes	Cold:	☐ No ☐ Yes
Hepatomegaly:	☐ No ☐ Yes	Mitral regurgitation:	☐ No ☐ Yes
Peripheral oedema:	☐ No ☐ Yes	Aortic stenosis:	☐ No ☐ Yes

5.4 Investigations/Procedures during hospitalisation

ECG: ☐ Performed ☐ Not performed

Rhythm: ☐ Sinus ☐ Atrial fibrillation/flutter ☐ Paced ☐ Other
Heart rate: |________| beats/min
QRS-duration: |________| ms
QT-duration: |________| ms
LBBB: ☐ No ☐ Yes
LVH: ☐ No ☐ Yes
Pathological Q-wave: ☐ No ☐ Yes

QTc-length automatic calculation
Bazett
Fridericia

Chest X-ray: ☐ Performed ☐ Not performed

Normal? ☐ No ☐ Yes

If No, please specify:

Cardiac enlargement:	☐ No ☐ Yes	Pulmonary congestion:	☐ No ☐ Yes
Alveolar oedema:	☐ No ☐ Yes	Other abnormality:	☐ No ☐ Yes

If Other, please describe: |________________________________|

Echo-Doppler: ☐ Performed ☐ Not performed

EF: |________| %
LVEDD: |________| mm
LVH: ☐ No ☐ Yes
E/A: |________| ratio
Deceleration time: |________| ms
LA measurement: ☐ Volume ☐ Dimension ☐ Unknown
IF Volume, LA Volume: |________| ml
IF Dimension, LA Dimension: |________| cm
Restrictive/pseudonormal pattern: ☐ No ☐ Yes ☐ Not evaluated
Mitral regurgitation moderate-severe: ☐ No ☐ Yes
Aortic stenosis moderate-severe: ☐ No ☐ Yes
Aortic regurgitation moderate-severe: ☐ No ☐ Yes
Tricuspid regurgitation moderate-severe: ☐ No ☐ Yes

Exercise test: ☐ No ☐ Yes ☐ Patient cannot do the test

Peak exercise, cycle ergometer: |________| watt
Peak exercise, treadmill: |________| metres
Peak VO2: |________| ml/kg/min
6 min walk test: |________| metres

Holter Monitoring: ☐ Performed ☐ Not performed

Mean HR: |________| beats/min
PVC hour: |________| complexes/24h
Unsustained VT: ☐ No ☐ Yes
Sustained VT: ☐ No ☐ Yes
Atrial fibrillation: ☐ No ☐ Yes

Coronary Angiography: ☐ Performed ☐ Not performed

Cardiac CT: ☐ Performed ☐ Not performed

PCI/CABG: ☐ Performed ☐ Not performed

EPS (Electrophysiological Study)**:** ☐ Performed ☐ Not performed

Inducible Sustained VT/VF: ☐ No ☐ Yes
Inducible Atrial fibrillation: ☐ No ☐ Yes
Major conduction abnormalities: ☐ No ☐ Yes

Transcatheter Ablation: ☐ Performed ☐ Not performed

Atrial: ☐ No ☐ Yes
Ventricular: ☐ No ☐ Yes
Nodal: ☐ No ☐ Yes

Electric cardioversion: ☐ Performed ☐ Not performed

Atrial Fibrillation ☐ No ☐ Yes
VT/VF: ☐ No ☐ Yes

Right Heart Catheterization: ☐ Performed ☐ Not performed

mPAP: |________| mmHg
Right atrial pressure: |________| mmHg
PCW: |________| mmHg
CI: |________| L/min/m²

Myocardial Scintigraphy: ☐ Performed ☐ Not performed

Resting ischaemia: ☐ No ☐ Yes
Myocardial viability: ☐ No ☐ Yes

Endomyocardial Biopsy: ☐ Performed ☐ Not performed

IAPB: ☐ Performed ☐ Not performed

CRT implantation: ☐ Not indicated ☐ Indicated ☐ Already implanted

If Indicated, treatment: ☐ Not planned ☐ Planned

If Not planned, reason:
☐ Absence of clinical indication
☐ Cost issues
☐ Patient refusal
☐ Logistic issues
☐ Other

ICD implantation: ☐ Not indicated ☐ Indicated ☐ Already implanted

If Indicated, treatment: ☐ Not planned ☐ Planned

If Not planned, reason:
☐ Absence of clinical indication
☐ Cost issues
☐ Patient refusal
☐ Logistic issues
☐ Other

Education: ☐ No ☐ Yes

If Yes, please specify:

Heartfailurematters.org:	☐ No ☐ Yes	Other web sites:	☐ No ☐ Yes
National booklets:	☐ No ☐ Yes	Other:	☐ No ☐ Yes

Rehabilitation: ☐ Performed ☐ Not performed

5.5 Chemistry at Hospital Discharge / Outcome

White blood cells:	|_______|	☐ Cells/microL<br>☐ Giga/L	Total cholesterol:	|_______|	☐ mg/dL<br>☐ mmol/L
Hemoglobin:	|_______|	☐ g/dL<br>☐ mmol/L<br>☐ g/L	Fasting glucose:	|_______|	☐ mg/dL<br>☐ mmol/L
S-creatinine:	|_______|	☐ mg/dL<br>☐ µmol/L	HbA1c:	|_______|	☐ %<br>☐ mmol/mol
Nitrogen measured by:	☐ BUN	☐ Urea	BNP:	|_______|	☐ pg/mL<br>☐ pmol/L
If BUN[9]:	|_______|	☐ mg/dL<br>☐ mmol/L	NT-proBNP:	|_______|	☐ pg/mL<br>☐ pmol/L
If urea:	|_______|	☐ mg/dL<br>☐ mmol/L	Sodium:	|_______|	mEq/L or mmol/L
Uric acid:	|_______|	☐ mg/dL<br>☐ µmol/L	Potassium:	|_______|	mEq/L or mmol/L
Proteinuria:	☐ No	☐ Yes	Bilirubin:	|_______|	☐ mg/dL<br>☐ µmol/L
TSH:	|_______|	mIU/L	HIV infection:	☐ No	☐ Yes
Troponin I or T:	|_______|	ng/mL or pg/mL	Hs-CRP:	|_______|	mg/L
Hs-Troponin I or T:	|_______|	ng/mL or pg/mL			

5.6 Scores

Was prognosis evaluated using a risk score? ☐ No ☐ Yes

If Yes,
- ☐ SEATLE
- ☐ CHARM
- ☐ GISSI-HF
- ☐ MAGGIC
- ☐ MECKI
- ☐ HF ACTIONS
- ☐ EMPHASIS
- ☐ OTHER

If Other, please specify: |______________________________|

References

- SEATLE : Wayne C; Levy et al – Circulation 2006, 113, 1424-1433
- CHARM : Stuart J. Pocock et al – European Heart Journal 2006, 27, 65-75
- GISSI-HF : Simona Barlera et al – Circulation Heart Failure 2013, 6, 31-39
- MAGGIC : Stuart J. Pocock et al – European Heart Journal 2012, 34, 1404-13
- MECKI : Piergiuseppe Agostini et al – Interventional Journal of Cardiology, 2012, 06-113
- HF ACTION: Christopher M. O'Connor et al - Circulation Heart Failure 2012, 5, 63-71
- EMPHASIS : Timothy J. Collier et al - European Heart Journal 2013, 34, 2823-9

Heart Failure Long-Term Registry

Sign off

6.1 CRF Completed

Answer YES to the question below to confirm that you have completed the questionnaire.

CRF Completed: ☐ No ☐ Yes

Heart Failure Long-Term Registry
12-Month Follow-Up

7.1 Status at 12 months post inclusion

Lost to follow-up: ☐ No ☐ Yes *If Yes, skip to section 7.4*

Contact date: |__/__/____| dd/mm/yyyy Contact method: ☐ Visit ☐ Phone

Where is data collected: ☐ Hospital ☐ Primary care ☐ HF clinic ☐ Other organisation

By whom: ☐ Cardiologist ☐ GP ☐ Internal medicine doctor ☐ Geriatrician
☐ Nurse ☐ Physiotherapist ☐ Palliative care nurse ☐ Other

Days hospitalised prior year: |____| days

Vital status: ☐ Alive ☐ Dead

If Dead:

Date of death: |__/__/____| dd/mm/yyyy

Site of death: ☐ Home ☐ In public area ☐ Nursing home
☐ Emergency room ☐ Hospital ☐ Unknown

Causes of death: ☐ Procedure related ☐ Non procedure related ☐ Unknown

Causes of death: ☐ Cardiac ☐ Vascular ☐ Non cardiovascular ☐ Unknown

If Cardiac causes, Mode: ☐ Sudden ☐ Non sudden
Cause: ☐ AMI ☐ Heart Failure ☐ Arrhythmia ☐ Other

If Vascular causes, Details:
☐ Ischemic stroke
☐ Hemorrhagic stroke
☐ Systemic hemorrhage
☐ Peripheral embolism
☐ Pulmonary embolism

If other cardiac causes, please specify: |____|

If Alive,

Heart rate: |______| beats/min

Blood pressure (systolic/diastolic): |_____| / |_____| mmHg

NYHA class: ☐NYHA I ☐NYHA II ☐NYHA III ☐NYHA IV

Re-hospitalisation (since discharge):

Re-hospitalisation		Date of Re-hospitalisation dd/mm/yyyy	Duration (days)	Primary Cause				
				Cardiac, non HF	HF	Vascular	Renal Dysfunction	Non CV
#1	☐ No ☐ Yes	|__/__/____|	|___|	☐	☐	☐	☐	☐
#2	☐ No ☐ Yes	|__/__/____|	|___|	☐	☐	☐	☐	☐
#3	☐ No ☐ Yes	|__/__/____|	|___|	☐	☐	☐	☐	☐
#4	☐ No ☐ Yes	|__/__/____|	|___|	☐	☐	☐	☐	☐
#5	☐ No ☐ Yes	|__/__/____|	|___|	☐	☐	☐	☐	☐

7.2 Chemistry

Blood test: ☐ Performed ☐ Not performed

White blood cells:	|_______|	☐ Cells/microL<br>☐ Giga/L	Total cholesterol:	|_______|	☐ mg/dL<br>☐ mmol/L
Hemoglobin:	|_______|	☐ g/dL<br>☐ mmol/L<br>☐ g/L	Fasting glucose:	|_______|	☐ mg/dL<br>☐ mmol/L
S-creatinine:	|_______|	☐ mg/dL<br>☐ µmol/L	HbA1c:	|_______|	☐ %<br>☐ mmol/mol
Nitrogen measured by:	☐ BUN	☐ Urea	BNP:	|_______|	☐ pg/mL<br>☐ pmol/L
If BUN(9):	|_______|	☐ mg/dL<br>☐ mmol/L	NT-proBNP:	|_______|	☐ pg/mL<br>☐ pmol/L
If urea:	|_______|	☐ mg/dL<br>☐ mmol/L	Sodium:	|_______|	mEq/L or mmol/L
Uric acid:	|_______|	☐ mg/dL<br>☐ µmol/L	Potassium:	|_______|	mEq/L or mmol/L
Proteinuria:	☐ No	☐ Yes	Bilirubin:	|_______|	☐ mg/dL<br>☐ µmol/L
TSH:	|_______|	mIU/L	HIV infection:	☐ No	☐ Yes
Troponin I or T:	|_______|	ng/mL or pg/mL	Hs-CRP:	|_______|	mg/L
Hs-Troponin I or T:	|_______|	ng/mL or pg/mL			

7.3 Medication (11)

Is the patient under any of the medication below? ☐ No ☐ Yes

CV Drugs *Doses should be total given in one day.*

Drug type	Generic name and daily Dose
ACE inhibitors	☐ No<br>☐ Ramipril<br>☐ Enalapril<br>☐ Perindopril<br>☐ Lisinopril<br>☐ Captopril<br>☐ Fosinopril<br>☐ Other<br>If No, ☐ Contraindicated ☐ Not tolerated ☐ Other<br>If Contraindicated, ☐ Bilateral renal stenosis<br>☐ Hyperkalemia<br>☐ Symptomatic hypotension<br>☐ Severe renal dysfunction<br>☐ Other<br>If Not tolerated, ☐ Cough<br>☐ Worsening renal function<br>☐ Symptomatic hypotension<br>☐ Hyperkalemia<br>☐ Angioedema<br>☐ Other<br>If Yes, Daily dose: |______| mg<br>Reason for target dose not reached ☐ Cough<br>☐ Worsening renal function<br>☐ Symptomatic hypotension<br>☐ Hyperkalemia<br>☐ Angioedema<br>☐ Still in uptitration<br>☐ Other
Angiotensin II receptor Blockers (ARB)	☐ No<br>☐ Candesartan<br>☐ Losartan<br>☐ Valsartan<br>☐ Other<br>If No, ☐ Contraindicated ☐ Not tolerated ☐ Other<br>If Contraindicated, ☐ Bilateral renal stenosis<br>☐ Hyperkalemia<br>☐ Symptomatic hypotension<br>☐ Severe renal dysfunction<br>☐ Other<br>If Not tolerated, ☐ Worsening renal function<br>☐ Symptomatic hypotension<br>☐ Hyperkalemia<br>☐ Angioedema<br>☐ Other<br>If Yes, Daily dose: |______| mg<br>Reason for target dose not reached ☐ Worsening renal function<br>☐ Symptomatic hypotension<br>☐ Hyperkalemia<br>☐ Angioedema<br>☐ Still in uptitration<br>☐ Other

Sacubitril / Valsartan<br><br>(ARNI: Angiotensin Receptor Neprilysin Inhibitor)	☐ No<br>☐ Yes<br><br>If No, ☐ Contraindicated ☐ Not tolerated ☐ Other<br>If Yes, Daily dose: |______| mg
Beta blockers	☐ No<br>☐ Carvedilol<br>☐ Bisoprolol<br>☐ Metoprolol<br>☐ Nebivolol<br>☐ Other<br><br>If No, ☐ Contraindicated ☐ Not tolerated ☐ Other<br>If Contraindicated, ☐ Asthma<br>☐ Bradyarrhythmia<br>☐ PAD<br>☐ Symptomatic hypotension<br>☐ Other<br>If Not tolerated, ☐ Broncospasm<br>☐ Worsening PAD<br>☐ Worsening HF<br>☐ Bradyarrhythmia<br>☐ Sexual dysfunction<br>☐ Symptomatic hypotension<br>☐ Other<br><br>If Yes, Daily dose: |______| mg<br>Reason for target dose not reached ☐ Broncospasm<br>☐ Worsening PAD<br>☐ Worsening HF<br>☐ Bradyarrhythmia<br>☐ Sexual dysfunction<br>☐ Symptomatic hypotension<br>☐ Still in uptitration<br>☐ Other
Mineralocorticoid receptor antagonists	☐ No<br>☐ Spironolactone<br>☐ Eplerenone<br>☐ Canrenone<br>☐ Other<br><br>If No, ☐ Contraindicated ☐ Not tolerated ☐ Other<br><br>If Contraindicated, ☐ Hyperkalemia<br>☐ Severe renal dysfunction<br>☐ Other<br>If Not tolerated, ☐ Hyperkalemia<br>☐ Worsening renal function<br>☐ Gynecomastie<br>☐ Other<br><br>If Yes, Daily dose: |______| mg<br>Reason for target dose not reached ☐ Hyperkalemia<br>☐ Worsening renal function<br>☐ Gynecomastie<br>☐ Still in uptitration<br>☐ Other

Diuretics: Oral	☐ No<br>☐ Bendrofluazide<br>☐ Chlorthalidone<br>☐ Hydrochlorothiazide<br>☐ Furosemide<br>☐ Indapamide<br>☐ Torasemide<br>☐ Bumetanide<br>☐ Other Daily dose: |______| *mg*
Diuretics oral (2nd medication)	☐ No<br>☐ Bendrofluazide<br>☐ Chlorthalidone<br>☐ Hydrochlorothiazide<br>☐ Furosemide<br>☐ Indapamide<br>☐ Torasemide<br>☐ Bumetanide<br>☐ Other Daily dose: |______| *mg*
Ivabradine	☐ No ☐ Yes If Yes, Daily dose: |______| mg<br>If No, ☐ Atrial Fibrillation/Flutter<br>☐ EF > 35 %<br>☐ HR < 70 bpm<br>☐ Medication still not available<br>☐ Not tolerated<br>☐ Other If Other: |__________________|
Digitalis	☐ No ☐ Yes
Statins	☐ No ☐ Yes
Antiplatelets	☐ No ☐ Yes
Anticoagulants	☐ No ☐ Yes
Amiodarone	☐ No ☐ Yes
Nitrates	☐ No ☐ Yes
Calcium channel blockers	☐ No ☐ Yes
Antiarrhythmics	☐ No ☐ Yes
Direct renin inhibitors	☐ No ☐ Yes

Non CV drugs:

Treatment for COPD	☐ No<br>☐ Corticosteroids<br>☐ Beta2 agonists<br>☐ Anticholinergic agents<br>☐ Xanthine agents
Anti-diabetic drugs: Oral	☐ Metformin<br>☐ Glitazones<br>☐ Incretins<br>☐ Sulphanylurea<br>☐ Other<br>☐ None
Anti-diabetic drugs: Insulin	☐ No ☐ Yes
Allopurinol	☐ No ☐ Yes
NSAIDs:	☐ No ☐ Yes
Antidepressants:	☐ No ☐ Yes
Number of non CV drugs:	|_____|

7.4 CRF Completed

Answer YES to the question below to confirm that you have finished the 12 month follow-up data collection for this patient. Only completed CRFs will be taken into consideration for the analysis.

CRF Completed: ☐ No ☐ Yes

Help

1	Outpatient = All patients seen in the outpatient clinic.<br>Hospitalised = All patients admitted for acute HF.
2	The date the patient was born as recorded on their birth certificate. Age should be greater than 18 years.
3	Ischaemic dilated cardiomyopathy should be classified as Ischaemic heart disease.<br>HFPEF Syndrome: one or more of hypertension, diabetes, obesity, older age, deconditioning, sleep apnea or others.
4	Patient currently enrolled in a randomised clinical trial? Will not affect enrolment as this is observational study.
5	Indicate if the patient has a history confirming any form of tobacco use in the past. This includes cigarettes, cigar and/or pipe.<br>Current = patient regularly smokes a tobacco product / products one or more times per day or has smoked in the 30 days prior to this admission.<br>Former = patient has stopped smoking tobacco products greater than 30 days before this admission.
6	Indicate if the patient has a history of hypertension diagnosed and/or treated by a physician
7	Serum creatinine >1.5 mg/dL
8	Viral, not alcoholic hepatitis
9	Blood urea nitrogen
10	Ventricular Tachycardia/Ventricular Fibrillation
11	Medications at hospital discharge/ambulatory visit
