# Supplementary material for: Patient, hospital and country-level risk factors of all-cause mortality among patients with chronic heart failure: Prospective international cohort study
Source: PLoS One. 2021 May 10;16(5):e0250931. doi: 10.1371/journal.pone.0250931 (PMC8109791; doi:10.1371/journal.pone.0250931)
Supplement: S2 File — (PDF) [file pone.0250931.s003.pdf]

Heart Failure Long-term Registry

Patient Information Sheet

You have been admitted to the hospital with the diagnosis of heart failure and your disease will be treated according to the best knowledge of the physician who takes care of you.

Heart failure is a medical condition resulting from different causes and is characterized by an inability of the heart to function correctly to pump blood to the body. Also, it is accompanied by metabolic and hormonal changes that may reflect the stage of the disease.

We would like to invite you to take part in a large international pilot registry organised by the European Society of Cardiology, called EURObservational Research Programme - Heart Failure Long-term Registry. This project aims to review the care that people similar to yourself are offered and receive whilst in hospital and how you feel 12 months later.

The purpose of this registry is to evaluate what sort of investigation and treatment patients with heart failure similar to yours receive in different countries and in different settings within Europe and in other countries in the world. The research will lead to practical recommendations to improve treatment of heart failure on local, national and international level.

It will not be necessary for you to undergo any medical tests or treatments extra to the normal care you would receive for your condition. Similarly, no treatments will be withheld as a result of you taking part in this research project. Your care will continue as normal under your doctor's expertise. Thus, you will not be exposed to any risks or hazards by taking part in this study.

We will collect data concerning your demography, past medical history and the treatment you receive as well as the diagnostic tests that are performed during your stay at the hospital and the treatment given to you.

All information collected about your medical condition will remain strictly confidential under the law of your country. These data will be identified by a confidential number code kept in your hospital, thus data transferred to the central database will be **anonymised**.

We would like to contact you once more after one year since enrolment. Then the local doctor or nurse will call you by telephone and ask you several questions about your current health condition and any medical events that may have occurred during this year.

If you need further information or have any questions about this study, please do not hesitate to contact the researcher or data collecting person in charge of this registry. Thank you for your help and assistance regarding this international study which we hope will enable improvement of the care of patients with heart problems in the near future.

If you do not wish to take part, you do not need to justify your decisions and this will not affect your usual care in any way.

Patient Consent

- I confirm that I have read the information form and that I understand the information. I have had the opportunity to ask additional questions and have had adequate replies to my questions and I have had enough time to consider participation.
- I am aware that my participation is completely voluntary.
- I authorize the following persons, namely: members of the medical research team; members of the ministry of public health; members of the medical ethics committee; local auditors or other competent authorities, to have access to my medical files and study data.
- I consent to the storage of my personal information for up to 15 years after completion of the study.
- I understand that I have absolute right to access, amend and oppose any use and record of this personal data by informing my doctor.
- I understand that the data obtained will only be used for scientific research purposes.
- I agree to the use of my data as explained in the Patient Information sheet and consent to participate in the Heart Failure Long-term Registry.

Date: ....

Patient Name: .... **Signature** ....

Investigator Name: .... **Signature** ....

Name of the person (medical doctor or research nurse), who informed the patient:

....

For further information please contact the researcher:

....

Note concerning your personal data

You have personal data which is, according to the French Law on data processing and Civil Liberties, number 78-17 of 6 January 1978, articles 38 to 40, registered with the ESC. The recipients of these data are the EORP staff and contractors, EORP committee members and the National Coordinator. You have the absolute right to access, amend and oppose any use and record of this personal data by contacting:

By email: eorp@escardio.org

In writing: EURObservational Research Programme
European Society of Cardiology
Les Templiers
2035 Route des Colles
CS 80179 BIOT
06903 Sophia Antipolis - France
