# Supplementary material for: Patient, hospital and country-level risk factors of all-cause mortality among patients with chronic heart failure: Prospective international cohort study
Source: PLoS One. 2021 May 10;16(5):e0250931. doi: 10.1371/journal.pone.0250931 (PMC8109791; doi:10.1371/journal.pone.0250931)
Supplement: Attachment — Submitted filename: Rebuttal letter.docx [file pone.0250931.s004.docx]

**Bold= Authors;** Plain=Reviewer/Editors

Additional Editor Comments:

The manuscript is very interesting and topic. However I have only a concern about the importance of HF-related precipitating factors. Please see and discuss Testa G et al. Precipitating factors in younger and older adults with decompensated chronic heart failure: are they different? J Am Geriatr Soc. 2013 Oct;61(10):1827-8.

**We thank the Associate Editor for this comment. We discussed the importance of HF-related precipitating factors in the discussion and discussed the suggested paper by Testa et al. The paragraph that we added is the following**

***“The fact that the study design had a mandatory follow-up visit at 12 months to collect information on morbidity and mortality represented a strength and unique opportunity to study survival with a relatively uniform follow-up time across countries. At the same time, however, we had no information on the time course of patients’ HF over this period. Not being able to monitor the HF progress, little we know on possible precipitating factors that may lead to decompensation during the follow-up, and are known to be different in young and older patients (Testa et al., 2013). Nevertheless, we accounted for patients’ clinical and medical history, partly accounting for those factors that may act as precipitators in the HF time course.”***

Review Comments to the Author

Reviewer #1: In the manuscript entitled Patient, hospital and country-level risk factors of all-cause mortality among patients with chronic heart failure: Prospective international cohort study, Pongiglione and co-authors report on patient-, hospital- and country-level characteristics associated with 1-year all-cause mortality among patients with chronic HF including geographic and hospital variation in mortality. On 9,277 patients with chronic HF in the prospective cohort study European Society of Cardiology Heart Failure Long Term, the Authors report an all-cause 1-year mortality rate, across 142 hospitals located in 22 countries, of 7.1% which varied between countries and hospitals. These mortality rates were directly associated with increasing age, diabetes, peripheral artery disease, higher NYHA class. Conversely, treatment with angiotensin-converting enzyme inhibitor and angiotensin receptor antagonists and being managed in an HF clinic were found protective against mortality. Interestingly, no other hospital-level characteristics, and no country-level healthcare characteristics were associated with 1-year mortality, suggesting that between-hospital variance might be crucial for HF patients’ outcomes.
Overall, this is a very interesting manuscript on a crucial topic in HF management. The aim is clear, data analysis is well conducted and reported, and results are well discussed. I consider the manuscript acceptable for publication as it stands.

**We thank the reviewer very much for her/his positive and supportive feedback.**

Reviewer #2: The Authors explored hospital and country-level characteristics associated with 1-year all-cause mortality among patients with chronic HF and investigates geographic and hospital variation in mortality. They studied 9,277 patients with chronic HF enrolled between May 2011 and November 2017 in the prospective cohort study European Society of Cardiology Heart Failure Long Term registry across 142 hospitals, located in 22 countries. The mean age of the selected outpatients was 65 years and the all-cause 1- year mortality rate per 100 person-years was 7.1 and varied between countries (median 6.8, IQR 5.6-11.2) and hospitals (median 7.8, IQR 5.2-12.4). Mortality was associated with age (incidence rate ratio 1.03, 95% CI 1.02-1.04), diabetes mellitus (1.37, 1.15-1.63), peripheral artery disease (1.56, 1.27- 1.92), New York Heart Association class III/IV (1.91, 1.60-2.30), treatment with angiotensin-converting enzyme inhibitor and angiotensin receptor antagonists (0.71, 0.57-0.87) and HF clinic (0.64, 0.46-0.89). No other hospital-level characteristics, and no country-level healthcare characteristics were associated with 1-year mortality, with case-mix standardised variance between countries being very low and higher for hospitals (0.372).
I find the study of great interest, data support conclusion even if I suppose a possible role of a selection bias due to the selection made by European Society of Cardiology in the selection of hospital. The involved hospital are of recognize high quality and specifically devoted to cardiovascular disease. This reflect the great Job made the ESC in the diffusion of cardiovascular knowledge all over the Europe.

**We thank the reviewer very much for her/his positive feedback. We included the comment on the ESC role in selecting hospitals in the discussion in the following paragraph:**

***(..) Moreover, the fact that the selection of hospitals was made by ESC guarantees a recognized quality of participating hospitals, but at the same time entails a possible selection bias.***

References:

**We added the reference**

**Testa G, Della‐Morte D, Cacciatore F, Gargiulo G, D'Ambrosio D, Galizia G, et al. Precipitating factors in younger and older adults with decompensated chronic heart failure: are they different?. Journal of the American Geriatrics Society. 2013 Oct;61(10):1827-8.**

**And amended those not compliant with Vancouver form as described in Plos submission guidelines**
